# Correction to “Rapid On‐Site Digital Histopatholocal Evaluation (RODE) on Endobronchial Ultrasound (EBUS) Mediastinal Lymph Node Cryobiopsy Sampling Using Confocal Laser Microscopy—A Proof‐Of‐Concept Study”

**DOI:** 10.1002/rcr2.70159

**Published:** 2025-03-17

H. K. Gonuguntla, B. P. Vidyasagar, M. Shah, S. B. Radia, S. Tripathi, and V. Poletti, “Rapid On‐Site Digital Histopatholocal Evaluation (RODE) on Endobronchial Ultrasound (EBUS) Mediastinal Lymph Node Cryobiopsy Sampling Using Confocal Laser Microscopy—A Proof‐Of‐Concept Study,” *Respirology Case Reports* 13, no. 2 (2025): e70103, https://doi.org/10.1002/rcr2.70103.

In the title “Histopatholocal” was incorrect. This should have read: “Histopathological”. The title should be corrected as follows:

Rapid On‐Site Digital Histopathological Evaluation (RODE) on Endobronchial Ultrasound (EBUS) Mediastinal Lymph Node Cryobiopsy Sampling Using Confocal Laser Microscopy—A Proof‐Of‐Concept Study

The authors apologise for this error.

